# Real-World Clinical Outcomes of Manual Versus Powered Endoscopic Staplers in Minimally Invasive Lung Cancer Surgery

**DOI:** 10.3390/jcm15176793

**Published:** 2026-09-01

**Authors:** Cosimo Lequaglie, Gabriella Giudice, Annalisa Carlucci, Roberto Cascone

**Affiliations:** Thoracic Surgery Unit, IRCCS Centro di Riferimento Oncologico della Basilicata, via Padre Pio, 1, 85028 Rionero in Vulture, Italy; cosimo.lequaglie@crob.it (C.L.); gabriella.giudice@crob.it (G.G.); annalisa.carlucci@crob.it (A.C.)

**Keywords:** lung cancer resection, stapler devices, VATS, oncology

## Abstract

**Objectives:** Manual surgical staplers remain widely used in minimally invasive lung surgery, while powered devices have been introduced to improve tissue compression, staple formation, and device handling. This study compared clinical outcomes associated with manual and powered endoscopic staplers in patients undergoing minimally invasive lung cancer surgery, with postoperative air leak as the main outcome of interest. **Methods:** We retrospectively reviewed 400 consecutive patients who underwent VATS segmentectomy or lobectomy for lung cancer between January 2023 and June 2025. After 20 exclusions, 380 patients were analyzed: 190 treated with manual staplers (Group A), representing a historical control cohort, and 190 with powered staplers (Group B). Postoperative air leak, operative time, intraoperative blood loss, postoperative complications, and length of stay were compared. Surgeon-perceived ergonomic benefit was assessed after each powered-stapler procedure using a dichotomous yes/no question. **Results:** Postoperative air leak occurred less frequently with powered than manual staplers (10.0% vs. 17.9%; *p* = 0.0382; OR = 0.510, 95% CI: 0.279–0.931). No patient developed prolonged air leak (>5 days) or required additional intervention for air leak. Operative time (65.32 ± 2.52 vs. 66.74 ± 3.07 min; *p* < 0.0001) and intraoperative blood loss (86.09 ± 4.12 vs. 88.3 ± 4.47 mL; *p* < 0.0001) were statistically lower with powered staplers, although absolute differences were small. No significant differences were observed in postoperative atelectasis or length of stay. All 190 surgeon assessments indicated a perceived ergonomic advantage with the powered stapler. **Conclusions:** Powered staplers were associated with a lower incidence of postoperative air leak. Operative time and blood loss were also statistically lower, although the absolute differences were of limited clinical relevance and did not translate into shorter hospitalization. Powered staplers were consistently perceived as ergonomically advantageous, although this assessment was subjective and non-validated. These findings warrant cautious interpretation given the retrospective, single-center design and historical-control comparison.

## 1. Introduction

Since their introduction, surgical staplers have largely replaced manual suturing techniques due to their efficiency in reducing operative time and improving tissue closure reliability.

Stapling devices play a key role in video-assisted thoracoscopic surgery (VATS) and in the resection of lung and bronchial tissue. However, one of the most frequent complications following pulmonary parenchymal resection is air leakage, which, when persisting for more than five days, is defined as a prolonged air leak (PAL). PALs are associated with increased length of hospital stay, higher patient morbidity and mortality, and greater healthcare costs.

Over the years, various measures have been implemented to reduce air leaks and blood and/or serum loss, with the goal of shortening postoperative hospital stay. Staple cartridges have been progressively modified in both design and staple height to improve tissue compression and sealing performance.

The design of staple cartridges has evolved over time, with modifications in both the geometry of the cartridge and the height of the staples.

In addition, many modern cartridges feature a smooth, tapered, and textured surface, with variable staple heights that gradually change from the center to the periphery to optimize tissue compression.

Various types of cartridge reinforcements have also been introduced to strengthen the staple line, particularly useful in emphysematous, inelastic, or inflamed lung tissues.

Similar attention has been devoted to refining the motion of the blade and the opening and closing mechanisms of the jaws during both the stapling and cartridge-loading phases. In recent years, powered staplers have been developed to reduce the manual firing force required, allowing for more precise and consistent tissue compression. These devices aim to minimize adverse events while providing optimal, automated adjustment for a reliable and effective staple line.

The rise of minimally invasive surgery has further increased the adoption of endoscopic staplers, which are indispensable for procedures performed through limited-access approaches.

Based on these developments, we sought to compare the use of manual staplers with powered staplers. Manual staplers remain widely used due to their efficiency and safety; however, powered staplers offer technological innovations that improve flexibility and performance, often at comparable costs.

In this study, we evaluated the clinical performance of manual and powered staplers in terms of ergonomics, device malfunctions, and staple line integrity on pulmonary parenchymal tissue, using three-dimensional imaging analysis and retrospective data review [1,2,3,4,5].

## 2. Materials and Methods

### 2.1. Study Design and Patient Characteristics

This retrospective cohort study was designed to compare the clinical outcomes of powered versus manual staplers in patients who underwent video-assisted thoracoscopic major lung surgery (VATS) as lobectomy or sublobar resection for lung cancer at our Referral Cancer Center. Clinical data from 400 consecutive patients who underwent VATS segmentectomy or lobectomy between 1 January 2023, and 30 June 2025, at the I.R.C.C.S. Referral Cancer Center of Basilicata (Rionero in Vulture, Italy) were reviewed. There were 20 cases excluded due to open conversion (*n* = 10), 6 cases because were procedures without general anesthesia (*n* = 6), and 4 cases not resected for non-lung cancer (*n* = 4). Preoperative assessments were performed to exclude surgical contraindications. A total of 190 patients were included in the manual stapler group (Group A), in which the Tri-Staple™ device (Medtronic, 710 Medtronic Parkway, Minneapolis, MN, USA) was used. In the powered stapler group (Group B), lung resections were performed in 190 patients using the IntoCare™ Powered Endoscopic Linear Cutting Stapler (Building 6, Xinhong Industrial Park, No. 9, West Suhong Road, Suzhou Industrial Park, Suzhou, China). This device allows one-handed operation with powered firing, which may facilitate stable tissue compression and consistent staple formation during endoscopic procedures. The allocation of patients to Group A or Group B was determined solely by the availability of the powered stapler at our center. Since the powered stapler was not available until January 2024, Group A represented a historical control cohort of consecutive patients treated with manual staplers, whereas Group B consisted of consecutive patients treated with the powered stapler after its introduction into clinical practice. All procedures were performed by the same surgical team to ensure technical consistency.

The inclusion criteria were: (1) sublobar resection or lobectomy for lung cancer; (2) patients aged 18 years old or above; (3) resections underwent under general anesthesia; and (4) no surgical contraindications in preoperative examinations.

We excluded from the study patients under 18 years old, patients with poor performance status and/or poor functional reserve in respiratory function tests, past history of pulmonary surgery, conversion to thoracotomy, pneumonectomy or associated resections.

No a priori sample size calculation was performed because of the retrospective design; all consecutive eligible patients treated during the study period were included. No post hoc power analysis was performed; instead, effect size estimates with 95% confidence intervals were reported for the main comparative outcomes to provide information on the magnitude and precision of the observed effects.

We analyzed age, gender, smoking history, comorbidity, preoperative albumine serum level, type of lung resection, operative time, serum and blood loss, air leaks, post-operative complications, post-operative length of stay, comparing the data between the two groups.

Surgeon-perceived ergonomic benefit was assessed after each procedure by asking the primary surgeon a dichotomous (yes/no) question: “Did you perceive an ergonomic advantage when using the powered stapler compared with the previously used manual staplers for this type of procedure?” This assessment was subjective and was not based on a validated ergonomic scale.

The main outcome of interest was the incidence of postoperative air leak. For the purposes of the present study, this data was recorded as a dichotomous variable (present/absent), irrespective of its magnitude or duration. Chest tube duration was recorded for all patients, and chest tube removal coincided with hospital discharge. Additional outcomes included operative time, intraoperative blood loss, postoperative complications, length of hospital stay, pulmonary function at three months, and surgeon-perceived ergonomic benefit.

The study was reported in accordance with the Strengthening the Reporting of Observational Studies in Epidemiology (STROBE) guidelines.

### 2.2. Surgical Procedure

All patients underwent uniportal or biportal video-assisted thoracoscopic surgery (VATS) for lung resection. Preoperative three-dimensional (3D) reconstruction was performed based on each patient’s high-resolution computed tomography (CT) and 18F-fluorodeoxyglucose Positron Emission Tomography (PET/CT). Mixed reality technology was employed to accurately localize the pulmonary nodule and the suspected metastatic lymph nodes.

Patients were placed in the lateral decubitus position on the unaffected side under general anesthesia. A double-lumen endotracheal tube was inserted, and one-lung ventilation of the contralateral lung was established.

The utility incision was typically made at the fourth intercostal space along the anterior axillary line for upper lobectomies, and at the fifth intercostal space for middle or lower lobectomies. During the procedure, the pulmonary tributary veins, arteries, and bronchus were carefully identified according to the preoperative 3D reconstruction and were subsequently dissected and divided in sequence.

The vein was first divided after proximal closure with a white stapler cartridge, followed by the artery, which was divided in a similar manner. The bronchus was then transected using a violet Tri-Staple™ cartridge (manual device) or a blue/green cartridge with the IntoCare™ Powered Endoscopic Linear Cutting Stapler™. The intersegmental or interlobar plane was identified using the modified inflation–deflation method. The fissures were divided and completed with black or blue/violet cartridges, according to the type of stapling device used. No reinforced cartridges were employed. The choice of cartridge we indicated for the various bronchovascular structures and for the parenchyma depended mainly on the experience of our individual center but may vary based on the surgeon’s intraoperative tissue analysis. Cartridge selection was based on the surgeon’s intraoperative assessment of tissue characteristics and was performed according to the same criteria in both groups. In the powered stapler group, the cartridge corresponding to the tissue thickness and compression range of the manual cartridge that would otherwise have been selected was used.

The surgical specimen was retrieved using a 12 mm × 330 mm, 1500 mL laparoscopic retrieval bag (Pushbag, MEDILINER Healthcare, San Gregorio, Reggio Calabria, Italy). When indicated, the specimen was submitted for intraoperative frozen-section examination after the tumor focus had been marked and the resection margins confirmed to meet oncological criteria.

At least three lymph nodes were dissected from each of three different mediastinal stations. At the end of the procedure, a single chest tube with multiple side holes extending to the apex was placed. The tube was connected to a digital drainage system to objectively monitor air and fluid leakage (Drentech™ PalmEvo, Redax^®^, Poggio Rusco, Italy).

### 2.3. Assessments

Baseline characteristics and perioperative data were collected, including patient age, sex, smoking history, tumor location and size, postoperative pathological diagnosis, operative time, intraoperative blood loss, duration of chest tube placement, length of postoperative hospital stay, postoperative complications, routine postoperative hematologic parameters, and pulmonary function indices at three months after surgery.

Chest tube removal was indicated when no pneumothorax or atelectasis was present on imaging, no air leakage was detected, minimal fluctuation of water was observed in the drainage system, the pleural effusion was not predominantly hemorrhagic, and the daily drainage volume was ≤250 mL.

Postoperative complications included atelectasis and pulmonary infection. Postoperative air leak was monitored separately using the digital drainage system. For clinical monitoring, air leakage was graded as follows: grade 0 = none, grade 1 = during coughing only, grade 2 = during deep inspiration, and grade 3 = continuous. However, for the purposes of the comparative analysis, postoperative air leak was analyzed as a dichotomous variable (present/absent), irrespective of grade. Prolonged air leak (PAL) was defined as an air leak persisting for more than 5 postoperative days.

### 2.4. Statistical Analysis

Statistical analyses were performed using IBM SPSS Statistics version 19.0 (IBM Corp., Armonk, NY, USA). The normality of continuous variables was assessed using the Shapiro–Wilk test, and homogeneity of variances was evaluated before selecting the appropriate statistical test. Normally distributed continuous variables were expressed as mean ± standard deviation (SD) and compared using Student’s *t*-test. Non-normally distributed continuous variables were expressed as median and interquartile range (IQR) and compared using the Mann–Whitney U test. Categorical variables were expressed as number and percentage and compared using the chi-square test or Fisher’s exact test, as appropriate. Effect sizes were calculated for between-group comparisons. For normally distributed continuous variables, effect sizes were expressed as Hedges’ g with 95% confidence intervals (CIs), whereas rank-biserial correlation coefficients (r_rb) with 95% CIs were used for non-normally distributed continuous variables, where applicable. For changes in pulmonary function from baseline to 3 months (ΔFEV1 and ΔFEV1% predicted), calculated as the 3-month value minus the preoperative value, between-group differences were assessed using the Mann–Whitney U test and quantified using the Hodges–Lehmann location shift estimate with 95% CI. For categorical outcomes, associations were expressed as odds ratios (ORs) with 95% CIs. Standardized mean differences (SMDs) were additionally calculated for baseline characteristics to assess between-group balance. A two-sided *p*-value < 0.05 was considered statistically significant.

## 3. Results

There were no significant differences statistically in general data between the two groups (*p* > 0.05, Table 1). Hypertension was the most frequent comorbidity (Group A: 82; Group B: 78), followed by chronic obstructive pulmonary disease (COPD) (Group A: 39; Group B: 43), diabetes mellitus (Group A: 20; Group B: 22), and previous malignancies (Group A: 25; Group B: 21).

The 380 operations were completed successfully. The type of resection was shown in Table 2.

No significant differences were noted in age, sex, comorbidity, post-operative atelectasis (9.3% vs. 10.7% *p* = 0.6480) and postoperative hospital stay (2.57 ± 0.54 days vs. 2.62 ± 0.73 days—*p* = 0.5273). Operative time was significative reduced in Group B versus Group A (66.74 ± 3.07 min vs. 65.32 ± 2.52 min—*p* <0.0001) as the serum/blood loss (88.3 ± 4.47 mL vs. 86.09 ± 4.12 mL—*p* = 0.0032) and in air leaks (17.9% vs. 10%—*p* = 0.0382) (Table 3). No patient required perioperative blood transfusion. Moreover, no patient in either group developed prolonged air leak (>5 days), and none required additional interventions for postoperative air leak, which resolved with conservative management and continued chest drainage.

There were no significant differences between the two groups in inflammatory indices, including white blood cell count, neutrophil count, and neutrophil percentage, measured before and after surgery.

In Group B, the primary surgeon reported a perceived ergonomic advantage with the powered stapler compared with the previously used manual staplers after all 190 procedures (190/190, 100%).

No statistically significant differences were observed between the two groups in preoperative or 3-month postoperative pulmonary function, including FEV_1_ and FEV_1_% predicted. However, the change in FEV_1_ from baseline to 3 months (ΔFEV_1_) was significantly greater in Group A than in Group B [median (IQR): −0.11 (−0.16 to −0.09) L vs. −0.09 (−0.10 to −0.04) L; Hodges–Lehmann estimate, −0.04 L (95% CI, −0.05 to −0.02); *p* < 0.0001]. In contrast, no significant between-group difference was observed in the change in FEV_1_% predicted [median (IQR): −2 (−2 to −1) percentage points in both groups; *p* = 0.168] (Table 4).

## 4. Discussion

Thoracic surgical procedures for lung cancer have progressively shifted from open thoracotomy to minimally invasive approaches, particularly VATS and uniportal VATS, aiming to reduce surgical trauma, enhance postoperative recovery, shorten hospital stay, and improve perioperative outcomes while preserving oncological radicality [5,6,7,8]. In parallel with the development of minimally invasive surgical approaches, considerable attention has been paid to the optimization of surgical instruments. Refinements in forceps, clamps, scissors, and staplers have enhanced the precision of tissue approximation and division in the pulmonary parenchyma, bronchi, and pulmonary vessels. Improvements in staple design, closure mechanisms, staple-line configuration, and the number of staple rows has contributed significantly to these advancements [9,10,11].

Nonetheless, common challenges persist across different stapling devices, particularly tissue-sealing failure resulting in postoperative air leak [12,13]. In the present study, the incidence of postoperative air leak was significantly lower in the powered stapler group than in the manual stapler group (10.0% vs. 17.9%; OR = 0.510, 95% CI: 0.279–0.931). This finding is consistent with previous studies suggesting that powered stapling technology may improve staple-line performance and reduce postoperative air leakage during pulmonary resection. Air leak remains a clinically relevant outcome after lung resection because its persistence may delay chest tube removal, prolong hospitalization, and increase postoperative morbidity [12,13]. However, in our cohort, none of the patients developed prolonged air leak lasting more than five postoperative days, and none required additional procedures or interventions for air leak, with all cases resolving with conservative management and continued chest drainage. Thus, although the difference in overall postoperative air leak incidence favored powered staplers, the clinical implications of this finding should be interpreted in the context of the absence of PAL or air-leak-related invasive interventions in either group.

Operative time and intraoperative blood loss were also statistically significantly lower in the powered stapler group; however, the absolute differences between groups were small (approximately 1.4 min and 2.2 mL, respectively). Previous comparative studies have reported potential advantages associated with powered stapling systems in operative efficiency and perioperative outcomes [14,15,16,17]. Nevertheless, the magnitude of the differences observed in our cohort requires cautious interpretation. Notably, no patient in either group required perioperative blood transfusion, indicating that the observed difference in blood loss did not translate into a measurable benefit in terms of transfusion requirements. Similarly, no statistically significant difference in postoperative hospital stay was observed between the two groups. Therefore, statistical significance should not be interpreted as necessarily indicating a clinically meaningful benefit, and the modest reductions in operative time and blood loss observed in the present study are unlikely, by themselves, to substantially affect perioperative outcomes.

Postoperative pulmonary function was also comparable between the two groups. No statistically significant differences were observed in FEV_1_ or FEV_1_% predicted either preoperatively or at the 3-month follow-up. However, the reduction in FEV_1_ from baseline to 3 months was statistically greater in Group A than in Group B, with a Hodges–Lehmann estimated between-group difference of only 0.04 L (95% CI, 0.02–0.05 L). Although statistically significant, the small absolute magnitude of this difference suggests limited clinical relevance and should be interpreted cautiously. Consistently, the change in FEV_1_% predicted did not differ significantly between the groups, with a median reduction of 2 percentage points in both groups. Taken together, these findings do not suggest a substantial difference in pulmonary functional recovery at 3 months between patients treated with manual and powered staplers, despite the statistically significant difference observed in the absolute change in FEV_1_.

The potential differences between manual and powered staplers should also be interpreted in the context of the mechanical interaction between the stapling device and the target tissue. Staple height is a key factor in achieving adequate tissue compression while minimizing postoperative air and fluid leaks. Color-coded cartridges correspond to different staple heights and intended ranges of tissue thickness; however, selection of the appropriate cartridge remains largely dependent on the surgeon’s intraoperative assessment and experience because precise objective measurement of tissue thickness is not routinely available [18]. Inappropriate cartridge selection may result in either insufficient or excessive tissue compression and potentially contribute to staple-line complications [19,20]. In the present study, the same subjective selection criteria were applied in both groups, and, when the powered device was used, the cartridge corresponding to the tissue thickness and compression range of the manual cartridge that would otherwise have been selected was chosen. Although this approach provided consistency between the two groups, some degree of operator-dependent variability cannot be excluded.

Technological developments have attempted to improve staple-line reliability through both cartridge design and reinforcement strategies. Smooth and tapered cartridges have been developed to optimize tissue compression and sealing, while graded compression systems, such as Tri-Staple™ technology, are designed to reduce mechanical stress on the tissue after closure [21]. Staple-line reinforcement using biological or synthetic buttressing materials has also been investigated as a strategy to reduce postoperative air leakage [22,23,24,25,26,27], particularly in patients at increased risk because of emphysema, advanced age, or chronic pulmonary disease. More recently, powered stapling systems have introduced controlled firing mechanisms and technologies designed to improve consistency during tissue transection [15,28]. These developments provide a plausible technological rationale for investigating whether powered devices may influence staple-line performance and postoperative outcomes, although device-related benefits remain dependent on tissue characteristics, cartridge selection, and surgical technique.

Powered staplers may also offer technical and ergonomic advantages through single-handed operation, allowing the surgeon to position the device and control jaw opening, closure, and firing using the same hand. Reduced hand and wrist movement during firing may theoretically improve stability and limit tissue traction. In the present study, all 190 assessments performed after powered-stapler procedures indicated a surgeon-perceived ergonomic advantage compared with previously used manual staplers. However, this finding should be interpreted cautiously because ergonomics was assessed using a subjective dichotomous question rather than a validated ergonomic instrument. Consequently, our results indicate consistent surgeon-perceived preference but cannot objectively quantify the magnitude of ergonomic benefit or demonstrate reductions in surgeon fatigue.

These technical and ergonomic features may potentially have economic implications by reducing personnel requirements, operative resource utilization, or costs associated with postoperative management [14,15,29]. However, no formal quantitative cost analysis was performed in the present study; therefore, no conclusions regarding the cost-effectiveness or economic advantage of powered staplers can be drawn from our data. Previous studies have specifically investigated the economic implications of powered stapling technology [14,15,29], but these findings cannot be directly extrapolated to our cohort.

The relevance of stapling technology may be particularly evident during anatomical segmentectomy, which is increasingly used in selected patients with early-stage lung cancer [30,31]. Unlike lobectomy, segmentectomy requires accurate identification and division of the intersegmental plane, which lacks a natural anatomical boundary. Staplers can facilitate division of this plane and provide simultaneous tissue compression and sealing, but their use may also impair re-expansion of adjacent pulmonary parenchyma and can be technically challenging when maneuvering angles are restricted, particularly during uniportal VATS [32]. These considerations underline the importance of device maneuverability, controlled tissue compression, appropriate cartridge selection, and reliable staple-line formation in contemporary minimally invasive lung resection.

This study has several limitations. First, its retrospective and single-center design limits the generalizability of the findings. Furthermore, the manual stapler group represented a historical control cohort, as the powered stapler became available at our center only from January 2024. Therefore, a potential temporal bias cannot be excluded, as changes in perioperative management or increasing surgical experience over time may have influenced the observed outcomes. However, all procedures were performed by the same surgical team throughout the study period, which may have partially limited variability related to surgical technique. Moreover, cartridge selection was based on subjective intraoperative assessment rather than objective tissue-thickness measurement. Although the same selection criteria were applied in both groups, some degree of operator-dependent variability cannot be excluded. In addition, the assessment of surgeon-perceived ergonomic benefit was subjective and based on a non-validated dichotomous question rather than on a validated ergonomic assessment tool. No formal quantitative cost analysis was performed; therefore, the potential economic implications of powered stapling technology could not be assessed in the present cohort. Finally, the relatively small absolute differences observed for some statistically significant outcomes, including operative time, intraoperative blood loss, and the change in FEV_1_ at 3 months, should be interpreted cautiously with regard to their clinical relevance. Future prospective, multicenter studies with larger patient populations and standardized outcome measures are warranted to confirm the observed clinical findings, better establish their clinical relevance, objectively assess potential ergonomic benefits using validated instruments, and determine through dedicated health-economic analyses whether any technical or clinical advantages of powered staplers translate into measurable cost savings.

## 5. Conclusions

In this retrospective single-center cohort, the use of powered endoscopic staplers during minimally invasive lung cancer surgery was associated with a lower incidence of postoperative air leak compared with manual staplers. Operative time and intraoperative blood loss were also statistically lower in the powered stapler group; however, the absolute differences were small, and their clinical relevance appears limited. No significant difference in postoperative length of stay was observed. Powered staplers were consistently perceived by the operating surgeon as providing an ergonomic advantage; however, this finding was based on a subjective, non-validated assessment and should therefore be interpreted cautiously. Overall, these findings suggest potential clinical and technical advantages of powered staplers, but their clinical relevance should be confirmed in prospective, multicenter studies using standardized clinical and ergonomic outcome measures.

## Figures and Tables

**Table 1:** Characteristics of the patients.

Characteristic	(Group A) N° 190	(Group B) N° 190	*p-Value*	*SMD*
**Age** (**years**)	58.95 ± 10.4	58.88 ± 10.29	0.9211	−0.010
**Gender** (**M**)	98 (51.5%)	95 (50%)	0.8374	−0.032
**Smoking history**	175 (92.1%)	181 (95.3%)	0.2917	0.130
**Comorbidity**				
Hypertension	82 (43.2%)	78 (41.1%)	0.7553	−0.043
Diabetes	20 (10.5%)	22 (11.6%)	0.8700	0.034
History of previous malignant tumors	25 (13.2%)	21 (11.1%)	0.6371	−0.065
COPD	39 (20.5%)	43 (22.6%)	0.7083	0.051
**Preoperative Albumin**	40.97 ± 2.59	41.01 ± 2.58	0.8898	0.004

**Table 2:** Type of lung resection.

	Manual Standard Stapler (Group A) N° 190	Powered Stapler (Group B) N° 190
**Segmentectomy**	**80**	**75**
S1	14	14
S3	13	12
S8	13	12
S10	18	15
S9 + 10	22	22
**Lobectomy**	**110**	**115**
RUL	23	21
ML	6	8
RLL	20	25
LUL	22	30
LLL	33	25
RMLL	6	6

Abbreviations: RUL, right upper lobe; ML, middle lobe; RLL, right lower lobe; LUL, left upper lobe; LLL, left lower lobe; RMLL, right-middle and lower lobe.

**Table 3:** Comparison of selected variables.

Variables	Group A (*n* = 190)	Group B (*n* = 190)	*p-Value*	*Effect Size*
**Operative time** (**min**)	66.74 ± 3.07	**65.32 ± 2.52**	**<0.0001**	g = 0.504 95% CI: −0.708 to −0.300
**Serum/Blood loss** (**mL**)	88.3 ± 4.47	**86.09 ± 4.12**	**<0.0001**	g = −0.513 95% CI: −0.717 to −0.309
**Air leaks**	34 (17.9%)	**19** (**10%**)	**0.0382**	OR = 0.510 95% CI: 0.279–0.931
**Complications**	14 (7.4%) atelectasis	17 (8.9%) atelectasis	0.7078	OR = 1.235 95% CI: 0.591–2.584
**Length of stay** (**days**)	2.57 ± 0.54	2.62 ± 0.73	0.5273	r_rb = −0.013 95% CI: −0.118 to 0.094

**Table 4:** Pulmonary function at baseline and 3-month follow-up.

Pulmonary Function	(Group A) N° 190	(Group B) N° 190	*Effect Estimate*	*95% CI*	*p-Value*
FEV1 (L)					
Preoperative	2.01 (1.87–2.17)	2.01 (1.87–2.14)	-	-	0.799
3 months	1.87 (1.75–2.04)	1.92 (1.79–2.06)	-	-	0.064
ΔFEV1 (3 months—preop)	−0.11 (−0.16 to −0.09)	−0.09 (−0.10 to −0.04)	HL: −0.04 L	−0.05 to −0.02	<0.0001
FEV1 (% predicted)					
Preoperative	87.5 (79–96)	87.5 (79–95)	-	-	0.563
3 months	85.5 (78–94)	82 (78–93.75)	-	-	0.663
ΔFEV1% (3 months—preop)	−2 (−2 to −1)	−2 (−2 to −1)	HL: 0 pp	0 to 0	0.168

Abbreviations: FEV1, forced expiratory volume in 1 s; IQR, interquartile range; HL, Hodges–Lehmann estimate; pp, percentage points.

## Data Availability

The raw data supporting the conclusions of this article will be made available by the authors, without undue reservation.
